# Error in Trial Registration Number

**DOI:** 10.1001/jamanetworkopen.2020.6497

**Published:** 2020-04-27

In the Original Investigation “Comparison of Lemborexant With Placebo and Zolpidem Tartrate Extended Release for the Treatment of Older Adults With Insomnia Disorder: A Phase 3 Randomized Clinical Trial,”^1^ published on December 27, 2019, there was an error in the Abstract. The EudraCT trial registration number given as 2015-001463-39 should have been 2015-004347-39. This article has been corrected.^1^
